# A Novel Method about the Representation and Discrimination of Traffic State

**DOI:** 10.3390/s20185039

**Published:** 2020-09-04

**Authors:** Junfeng Jiang, Qiushi Chen, Jie Xue, Haobo Wang, Zhijun Chen

**Affiliations:** 1 College of artificial intelligence, Wuhan Technology and Business University, Wuhan 430073, China; jiangjunfeng@wtbu.edu.cn; 2 Intelligent Transportation Systems Center (ITSC), Wuhan University of Technology, Wuhan 430000, China; cqs@whut.edu.cn (Q.C.); whb150181393@whut.edu.cn (H.W.); chenzj556@whut.edu.cn (Z.C.); 3 Faculty of Technology, Policy and Management, Safety and Security Science Group (S3G), Delft University of Technology, 2628BX Delft, The Netherlands

**Keywords:** traffic state, K-means, multi-layer perceptron (MLP), road safety, traffic accidents, traffic congestion, traffic flow

## Abstract

The representation and discrimination of various traffic states play an essential role in solving traffic accidents and congestion as the foundation of traffic state prediction. However, the existing representation of the traffic state usually only considers the road congestion layer and divides the traffic state into congested and unblocked. Representation only at the congestion layer is difficult to reflect the road traffic state comprehensively. Therefore, we select three indicators from the layers of road congestion, road safety, and road stability, respectively, then utilizing K-means to cluster the traffic state. The clustering results can be regarded as a new type for the representation of a traffic state. As a result, the traffic states are divided into four classes, which comprehensively reflects the level of road congestion, safety, and stability. Using the four traffic states obtained from the clustering results as class labels, we applied a multi-layer perceptron (MLP) to classify the different traffic states, and the receiver operating characteristic (ROC) curve is assessed to verify the superiority of the classification results. Finally, a visual display of the real-time traffic state in a city’s central area was given.

## 1. Introduction

Traffic accidents and congestion have severely affected economic development and people’s travel efficiency, and thus need to be resolved [1,2]. Accurate road traffic state prediction is the key to solving these problems [3,4]. Nevertheless, as the basis of traffic state prediction, the representation and discrimination of the traffic state are still inconclusive. With the continuous improvement of intelligent transportation systems (ITS) and the constant optimization of traffic information collection technology, the channels of obtaining traffic information are more and more diverse, and the traffic information can be acquired by the detection equipment installed in the road network; the detection equipment mainly includes induction coils, radar, ultrasonic, etc. [5,6]. With the rapid development of video digitization, nowadays, video capture is more widely used [7,8], which provides support and guarantees the representation and analysis of traffic states.

In terms of the traffic state representation, scholars and many urban traffic management departments have proposed multiple representation methods for the traffic state of the road network. One approach is to use a single indicator to represent the traffic state, such as speed, traffic volume, traffic density, and vehicle spacing. For example, Khan et al. combined connected vehicles technology and artificial intelligence to form a CVT-AI model to process the data and evaluated the traffic state through traffic density [9]. Shi et al. collected GPS tracking data from GPS-equipped vehicles in real-time, then calculated the estimated spatial mean speed (eSMS), and converted the eSMS into a smoothing indicator to represent the traffic state [10]. Similarly, Tao et al. also employed speed as an indicator to represent the traffic state, and they aggregated the tracking data of various detectors, then obtained the average road link speed, and classified the average speed into a different traffic state level [11]. Hu et al. formed a value function-based urban traffic congestion measurement model. The model can better reflect the actual situation and has a good prospect of application [12]. Seo et al. believed that the spacing data (i.e., the distance between two vehicles) is closely related to traffic density. Their study assumed that there was a new type of rover, which can observe the distance between two vehicles. Then, the traffic state was estimated based on spacing data [13]. In addition, Wan et al. used the average deceleration duration under different traffic states as a key indicator to help predict the transition of the traffic state near the expressway merge bottleneck [14].

Because employing a single indicator to represent the traffic state is not comprehensive and objective enough, some scholars have studied another approach to represent the traffic state, which is to use multiple indicators to represent the traffic state. Antoniou et al. defined a traffic state in their research as the state of traffic at any given time, which can be described by multiple parameters such as traffic flow, traffic density, and speed [15]. Wang et al. proposed a real-time estimation method of traffic state based on the extended Kalman filter. Three indicators, namely traffic flow, average speed, and traffic density, were selected as the representation parameters. On this basis, the extended Kalman filter method was utilized to design the traffic state estimator [16]. Wang et al. instructed the test vehicle to drive on the actual road, and obtained data through the communication between the vehicle and the roadside unit, then selected the average travel time, the number of parking times, and the parking time to evaluate the road traffic state [17]. Xu et al. defined the road traffic state as the variables used to capture the road traffic behavior, including speed, traffic flow, queue length, and occupancy rate. After extracting the representative data, the road traffic running characteristic reference sequences (RTRCRS) were built to predict the traffic state [18]. Unlike representing the traffic state through traffic flow data, Zhan et al. used license-plate recognition data for the estimation and prediction of the traffic network state [19]. Cheng et al. proposed a new classification indicator, that is, the adequacy of the road network. They classified the traffic state based on the adequacy, which includes traffic flow, speed, and occupancy [20]. 

In addition, after representing the road traffic state, it is critical to building a suitable method to realize the discrimination of the traffic state. This can provide travelers with high-quality information services by achieving the real-time discrimination of the traffic state. With the development of information-processing technology, more and more intelligent technologies, such as fuzzy theory [21,22], artificial neural networks [23,24] and support vector machines [25,26] are being used in the study of traffic state and the prediction research of traffic state. The research on road traffic state is mainly based on the study of Automatic Incident Detection (AID). Fuzzy theory is proved to be able to solve the problem of fuzziness and uncertainty and provide theoretical support for the research of an autonomous driving system [27,28]. Hawas proposed a fuzzy system-based AID algorithm for urban arterial roads, and verified that the algorithm can better detect urban road traffic incidents [29]. Yuan et al. presented an AID algorithm based on the support vector machine (SVM), which utilizes the functional capabilities of SVM to achieve a nonlinear classification for traffic incident detection [30]. Ritchie et al. applied artificial neural networks (ANNs) in the study of the AID algorithm [31]. Chen et al. realized the discrimination of the traffic congestion state based on the temporal and spatial correlation, and then constructed a method to predict the urban road traffic state through an improved random forest algorithm [32]. Studies have shown that ANN has sound effects in the field of traffic state discrimination. The multi-layer perceptron (MLP) has strong nonlinear mapping capabilities, which can process a large amount of complex data and adjust the weights in the network. It has strong adaptive and self-learning capabilities. Therefore, we built a traffic state discriminator based on MLP in this paper. The main innovations of this paper are summarized as follows:

- Since the existing research only divides the road state from the perspective of whether the road is congested, it cannot reflect the comprehensive road traffic conditions well. In this paper, we selected the indicators from the layers of road congestion, road safety, and road stability, respectively, to represent various traffic states.
- On the basis of selecting multi-layer representation indicators, we applied the K-means clustering algorithm to divide the traffic state into four classes to achieve the representation of the traffic state.
- We used the traffic states obtained by K-means as class labels and built a traffic state discriminator based on MLP to realize an accurate discrimination of the traffic state.
- Finally, we visualized the data of a certain central urban area in accordance with the proposed traffic state representation and discrimination method.

The rest of the paper is organized as follows: the next section introduces our materials and methods, including our datasets, representation method, and discrimination method. In Section 3, we give the experimental process and results. The discussion of our research and future work is detailed in Section 4. Finally, the conclusions are addressed.

## 2. Materials and Methods

### 2.1. Datasets

In order to study the representation and discrimination of the traffic state, the full desensitization data of a main road in a central urban area of China was used in our experiment. These data were provided by the OpenITS Alliance. After collecting the original data on the virtual traffic measurement platform, we carried out abnormal data identification, abnormal data repair, and data standardization. The main fields of the dataset are shown in Table 1.

### 2.2. The Representation of the Traffic State

The purpose of the road traffic state representation is to provide timely and effective traffic information for traffic management departments to control traffic and for travelers to make travel plans.

#### 2.2.1. Selection of Representation Indicators

Many fundamental indicators can reflect the road traffic state, and different indicators correspond to different state layers. We selected three state layers and selected an evaluation indicator for each layer to achieve the representation and discrimination of the comprehensive traffic state of the road.

- Road congestion layer

The "Urban Road Traffic Operation Evaluation Indicator System", issued by Beijing, contains six evaluation indicators: road traffic performance index (TPI), road traffic congestion rate, congestion mileage ratio, congestion duration, frequent congested road sections, and the travel time reliability index, respectively; among them, TPI is the most widely used.

In the calculation of TPI, the traffic state of each road section is classified into five levels, which are unblocked, basically unblocked, lightly congested, moderately congested, and severely congested, respectively. Specific steps are as follows:

***Step 1:*** Calculate the time–mean–speed (V in Table 2) of each road section in the road network at an interval of no more than 15 minutes;

***Step 2:*** Determine the traffic state level of different road sections according to Table 2.

Therefore, we also chose the time–mean–speed as the evaluation indicator of the road congestion layer. Time–mean–speed (${\bar{v}}_{t}$) refers to the arithmetic average of the speeds of all vehicles passing through a road section in a certain period. It is often used to evaluate the degree of congestion on the road and is one of the essential parameters that represent the traffic state in the statistical time interval [33,34,35]. The calculation equation is as follows:

(1) $$\begin{array}{c} {\bar{v}}_{t}\;=\;\frac{1}{n}\overset{n}{\underset{i=1}{\sum }}v_{i} \end{array}$$

where n represents the total number of vehicles observed in a certain period, and $v_{i}$ represents the speed of the ith vehicle.

- Road safety layer

Space–mean–speed (${\bar{v}}_{s}$) refers to the average speed distribution of all vehicles driving within a certain length of road at a certain moment. When the observation length is a constant, its value is the harmonic average of the observed vehicle speed. The equation is as follows:

(2) $$\begin{array}{c} {\bar{v}}_{s}\;=\;\frac{1}{\frac{1}{n}\sum_{i=1}^{n}\frac{1}{v_{i}}}\;=\;\frac{ns}{\sum_{i=1}^{n}t_{i}}\; \end{array}$$

where s represents the length of the road section, $t_{i}$ denotes the travel time of the ith vehicle, n represents the number of times that vehicles travel the length s; $v_{i}$ refers to the travel speed of the ith vehicle.

When the vehicles have the same speed, the time–mean–speed is nearly equal to the space–mean–speed; otherwise, they have the following relationship:

(3) $$\begin{array}{c} {\bar{v}}_{s}={\bar{v}}_{t}-\frac{\sigma_{t}^{2}}{{\bar{v}}_{t}} \end{array}$$

where $\sigma_{t}$ represents the mean square deviation of the time–mean–speed observations. Based on the relationship between the time–mean–speed and the space–mean–speed, the variance of the time–mean–speed observation value can be deduced as:

(4) $$\begin{array}{c} \sigma_{t}^{2}=\left({\bar{v}}_{t}-{\bar{v}}_{s}\right)\times {\bar{v}}_{t} \end{array}$$

The variance of the time–mean–speed observation value represents the deviation of the time–mean–speed on the road, which can reflect the discrete situation of the vehicle speed distribution on the road, and thus reflect the road safety level.

- Road stability layer

In urban road traffic, the mixing of large vehicles will significantly reduce the speed of regular vehicles, and it will cause mutual interference between vehicles and increase the gap between vehicles, which may result in a waste of road resources and a decline in traffic capacity. Therefore, large vehicles are the principal objects of traffic control, and the ratio of large vehicles (e.g., large ration, LR) can reflect road stability and provide information support for traffic management departments to implement traffic control.

LR refers to the proportion of large vehicles in the total number of vehicles, which reflects the composition of various vehicles, and is an evaluation indicator of road traffic conditions. The calculation equation is as follows:

(5) $$\begin{array}{c} LR=\frac{N_{Large}}{N_{Total}} \end{array}$$

where $N_{large}$ refers to the number of large vehicles, which means the number of large vehicles passing the road; $N_{Total}$ refers to the total number of vehicles, which means the total number of vehicles crossing the road at a certain time.

#### 2.2.2. Traffic State Representation Based on K-Means

In order to achieve a multi-dimensional macroscopic traffic state representation, we employed the above three indicators to cluster the traffic state. Since the evaluation of the traffic state by the value of a specific indicator is subjective and ambiguous, and apart from that, the adjacent traffic state levels are closely connected, and there is no clear dividing line. In addition, for the same value, different people may divide it into different states. Therefore, our experiment clusters a large number of traffic evaluation indicators’ data based on the K-means clustering algorithm to achieve a reasonable representation of the traffic state.

K-means is an unsupervised clustering algorithm [36]. Its main idea is calculating the distance between samples for a given sample set according to a specific distance calculation method. Based on this distance, the sample set is divided into k clusters, so that the points within the cluster are as close as possible, and the points between the clusters are as far away as possible. Assuming that the sample is divided into k classes $\left(C_{1},C_{2},\ldots ,C_{k}\right)$, the goal of the algorithm is to minimize the square error E of the points within each cluster. E is calculated as shown in Equation (6):

(6) $$\begin{array}{c} E=\overset{k}{\underset{i=1}{\sum }}\underset{x\in C_{i}}{\sum }||xE-\mu_{i}||{}^{2} \end{array}$$

where x is the cluster sample object, and $\mu_{i}$ denotes the mean value of the data points in the cluster $C_{i}$.

The specific process of using K-means to cluster road traffic state evaluation indicators’ data is as follows:

(a) Input a sample set of road traffic state evaluation indicators and set the number of clusters as k;
(b) Select k data from the sample randomly as the mean of the initial cluster;
(c) Divide all objects in the dataset into the clusters represented by the nearest average point according to the current cluster average;
(d) Calculate the average central value points of the new clusters repeatedly until the average values have no changes;
(e) Output the clustering results of the k traffic states.

#### 2.2.3. Traffic State Discrimination Based on MLP

After classifying the traffic state by the K-means algorithm, a suitable classifier is needed to distinguish the traffic state of the new dataset. The real-time discrimination of the traffic state can provide travelers with high-quality information services. When building a traffic state classifier, we consider that the size of traffic datasets will continue to accumulate with the increase in traffic flow data, which may result in an increase in data dimensions. At the same time, due to the instability of traffic flow, the iterative update of datasets will company with the mixed abnormal data. All the above problems will reduce the accuracy of traffic state classification. However, MLP has strong nonlinear mapping capabilities and can handle a large amount of complex data. It has a very strong self-adaptation and self-learning ability and is also tolerant of abnormal data. Therefore, we built a traffic state classifier based on MLP.

**Figure 1:**
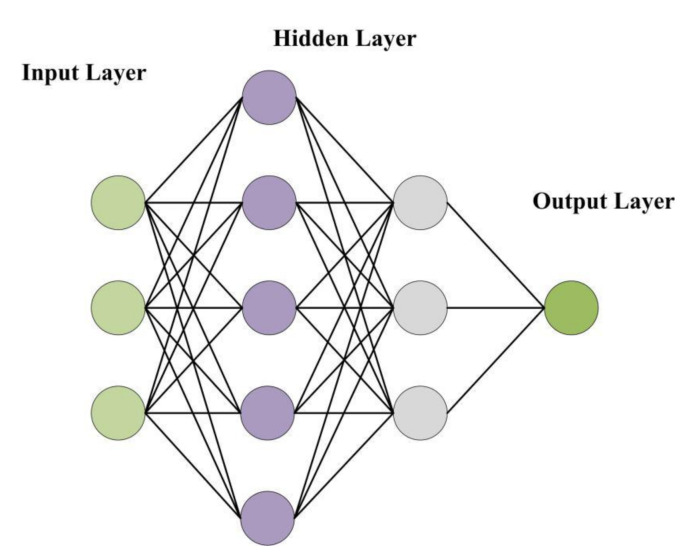
The network structure of multi-layer perceptron (MLP).

MLP [37] can map a set of input vectors to a set of output vectors. It is a neural network with a feedforward structure. In the network structure, MLP uses the most typical three-layer structure, which is composed of the input layer, hidden layer(s), and the output layer. The network structure is shown in Figure 1. Here is a network structure with two hidden layers.

The training process of MLP includes forward propagation and backpropagation. The calculation of forwarding propagation is shown in the following equations:

(7) $$\begin{array}{c} h_{j}=\overset{M}{\underset{i=0}{\sum }}w_{ij}x_{ij} \end{array}$$

(8) $$\begin{array}{c} a_{j}=g\left(h_{j}\right)=g\left(\overset{M}{\underset{i=0}{\sum }}w_{ij}x_{ij}\right) \end{array}$$

(9) $$\begin{array}{c} y=a_{k}=g\left(h_{k}\right)=g\left(\overset{M}{\underset{i=0}{\sum }}w_{jk}x_{jk}\right) \end{array}$$

where i represents the subscript of the previous layer of neurons or the input layer node; j represents the subscript of the current layer of neurons or the hidden layer node; k represents the subscript of the next layer of neurons, or the output layer node; $w_{ij}$ denotes the weight of each neuron in the previous layer to the current neuron; $w_{jk}$ is the weight of the current neuron to each neuron in the next layer; x represents the input. Equation (7) shows the process of weighted summation, where $h_{j}$ represents the weighted sum of all inputs of the current node. In Equation (8), $a_{j}$ represents the output value of the hidden layer neural unit, and $g\left(\;\right)$ is an activation function, usually using Sigmoid, Tanh, or ReLu activation function in MLP. Equation (9) shows the calculation formula of the output layer, where y represents the value of the output layer. In the classification task, the activation function here usually adopts the Softmax function.

After the basic model is built, the model parameters are updated during the training process. Due to the multi-layer network structure, it is impossible to directly use the loss to update the parameters of the middle layer. Still, the backpropagation of the loss from the top layer to the bottom layer can be applied to estimate the parameters. The measurement of loss often uses the sum of squares errors. The calculation of the loss function E is shown in Equation (10):

(10) $$\begin{array}{c} E=\frac{1}{2}\overset{N}{\underset{k=1}{\sum }}{\left(y-t\right)}^{2} \end{array}$$

where y is the output value of the model, t represents the true value of the training sample, and N represents the number of samples. The gradient descent method is widely used to find the optimal solution. In this way, backpropagation can realize the weight update of the middle layer of the network.

**Figure 2:**
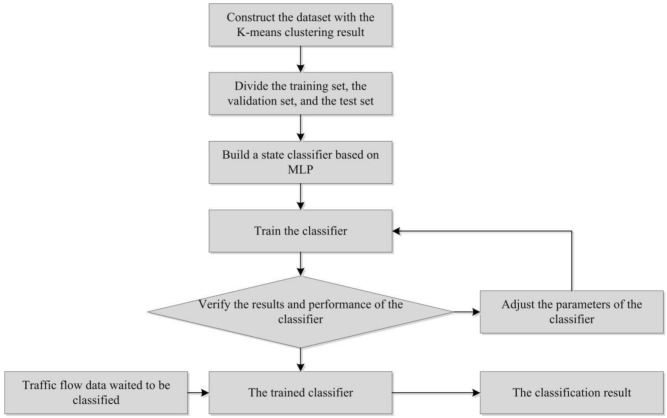
The experiment process of traffic state classification.

The main experimental steps of constructing MLP traffic state classifier in this paper are as follows, and the experimental process is shown in Figure 2.

(a) Construct the dataset: use the arithmetic mean vehicle speed, the variance of the time–mean vehicle speed observation value, and the large ratio as the input part of the model, and the K-means clustering result as the corresponding class label;
(b) Divide the training set, the test set and the validation set: the validation set and the test set each account for 10% of the total data;
(c) Build a state classifier based on MLP: the classifier includes an input layer, two hidden layers, and an output layer. The number of hidden units in the two hidden layers are 64 and 16, respectively. The hidden layer applies the ReLu function as the activation function; the output layer uses the Softmax function;
(d) Train the classifier and determine the hyperparameters of the model: set the initial learning rate, the number of iterations and the number of hidden layer units, and draw a graph of the loss function and the accuracy of the prediction result based on the training results. The receiver operating characteristic (ROC) curve graph of the classification result is obtained. Judge the fit degree of the model according to the graphs, and then adjust the hyperparameters.
(e) Input the test set to the trained model to obtain the classification result.

## 3. Results.

### 3.1. Evaluation Indicators of the Clustering Result

Although the K-means clustering algorithm has good results in processing extensive data and has been widely used, there are still some shortcomings. The value of parameter k in the K-means is very critical and will directly affect the clustering results. Specifying the value of k based on experience or the understanding of the dataset usually cannot obtain the optimal results. When the value of k is too small, it will cause the difference of the data in a cluster to become larger, which cannot reflect the difference between the real data; when the value of k is too large, it will cause the data of different clusters to have similar characteristics, which violates the goal of clustering.

To this end, the researchers explored many ways to determine the best k value. Based on the “principle of optimal clustering quality”, a principle which hopes that the distance between the elements in the cluster is the smallest, and the distance between the clusters is the largest, the Calinski–Harabasz (CH) and the silhouette coefficient (SC) are proposed to determine the clustering quality. The research results of many scholars show that they all have excellent judgment effects [38,39]. The calculation of CH is shown in Equation (11):

(11) $$\begin{array}{c} s\left(k\right)=\frac{tr\left(B_{k}\right)}{tr\left(W_{k}\right)}\frac{m-k}{k-1} \end{array}$$

where m is the number of samples in the training set, k is the number of divided clusters, $B_{k}$ is the covariance matrix between the different clusters, $W_{k}$ is the covariance matrix of the data between the same clusters, and tr is the trace of the matrix. The smaller the covariance of the data in the same cluster, the higher the similarity of the data in the same cluster; the larger the covariance between the different clusters, the greater the difference in data between the different clusters. Thus, the higher the value of the CH score, the greater the clustering effect, which means that the data similarity within a cluster is high, and the data difference between the different clusters is large.

The calculation of SC is shown in Equation (12):

(12) $$\begin{array}{c} S\left(i\right)=\frac{b\left(i\right)-a\left(i\right)}{max\left\{a\left(i\right),b\left(i\right)\right\}}\; \end{array}$$

where $a\left(i\right)$ is the average distance from sample i to the other sample points in the cluster, and $b\left(i\right)$ is the average distance from sample i to all the points in the nearest cluster, so the value range of the silhouette coefficient is [−1,1]. The larger the value, the better the clustering effect. If the value is negative, it indicates that the sample is classified into the wrong cluster.

Therefore, the CH and SC are selected to evaluate the rationality of the traffic state clustering results in this paper, and multiple sets of k values are set to classify the traffic state. The experimental results and the representation of the traffic state are shown in Section 3.2.

### 3.2. Clustering Results

The dataset was reconstructed based on the indicators selected in Section 2.2.1 to obtain the traffic state evaluation indicators’ dataset. We established a traffic state classification model based on K-means to achieve traffic state classification. The main experimental steps are as follows:

(a) Make a sample set: select 3,1968 traffic flow data with a sampling interval of 5 minutes in the central urban area and calculate the three traffic state evaluation indicators mentioned in Section 2.2.1 for each original sample. Three traffic state evaluation indicators constitute a clustering sample dataset;
(b) Construct a traffic state clustering model based on K-means, and set multiple sets of k values, cluster the clustering sample dataset and obtain multiple clustering results;
(c) The CH and SC are employed as the evaluation indicators and the final clusters k which is most suitable for the division of the sample set is selected according to the score of the CH and SC;
(d) Analyze the traffic states based on the optimal k-value clustering result.

**Figure 3:**
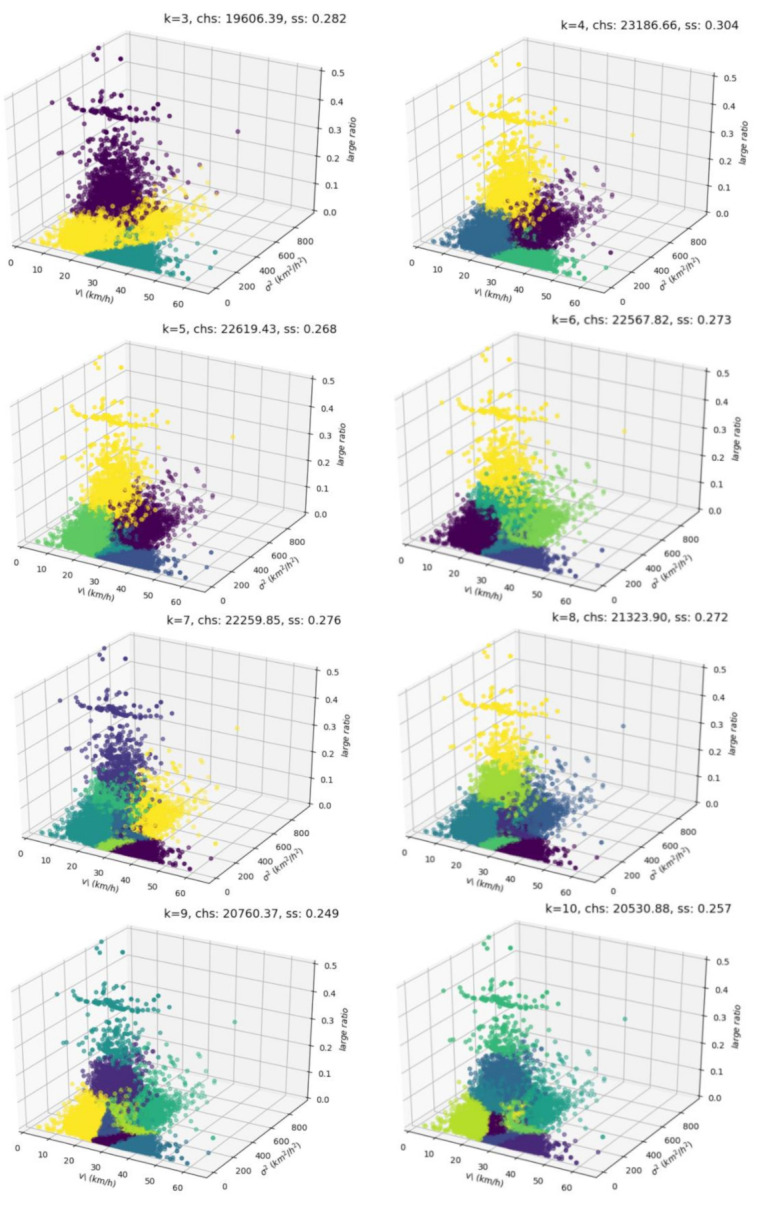
Clustering results of the traffic state under the different k

Through the above traffic state clustering experiment, this paper sets the parameter k as 3, 4, 5, 6, 7, 8, 9, and 10 in the verification of the optimal cluster number k. The results of eight clustering samples are shown in Figure 3.

**Figure 4:**
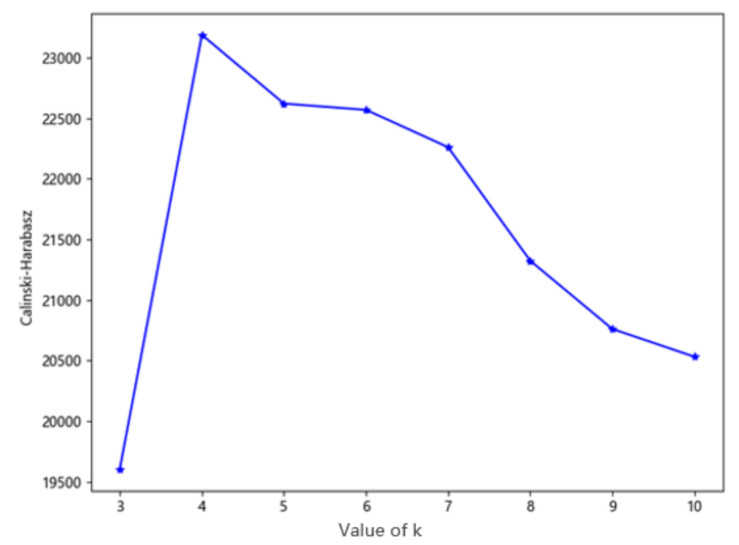
The Calinski–Harabasz (CH) score under different k

**Figure 5:**
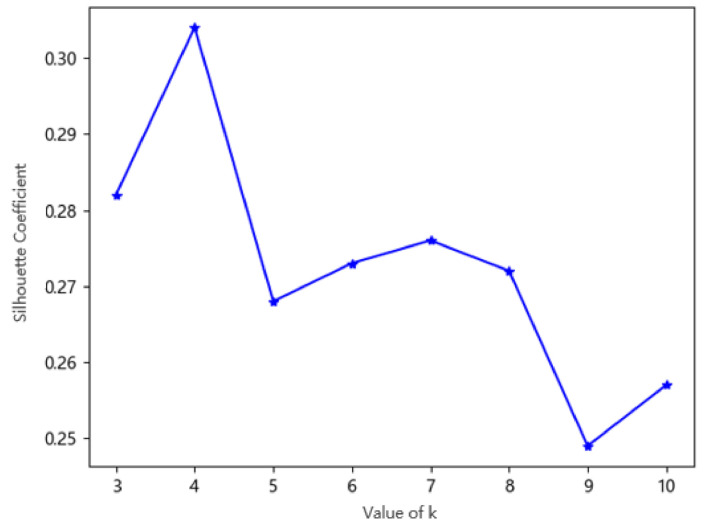
The silhouette coefficient (SC) score under the different k

According to the clustering results of different k values, the above eight groups of experiments were verified based on the CH and SC. The number of clusters with the highest score of the CH and SC was selected as the value of k in this experiment. The eight clustering results corresponding to the CH and SC scores are shown in Figure 4 and Figure 5. It can be concluded from the evaluation results that when the value of k is 4, both the CH and SC score are the highest. At this time, the data in the same cluster has little difference, and the data in different clusters has significant differences, that is to say, the clustering result is the most reasonable.

**Figure 6:**
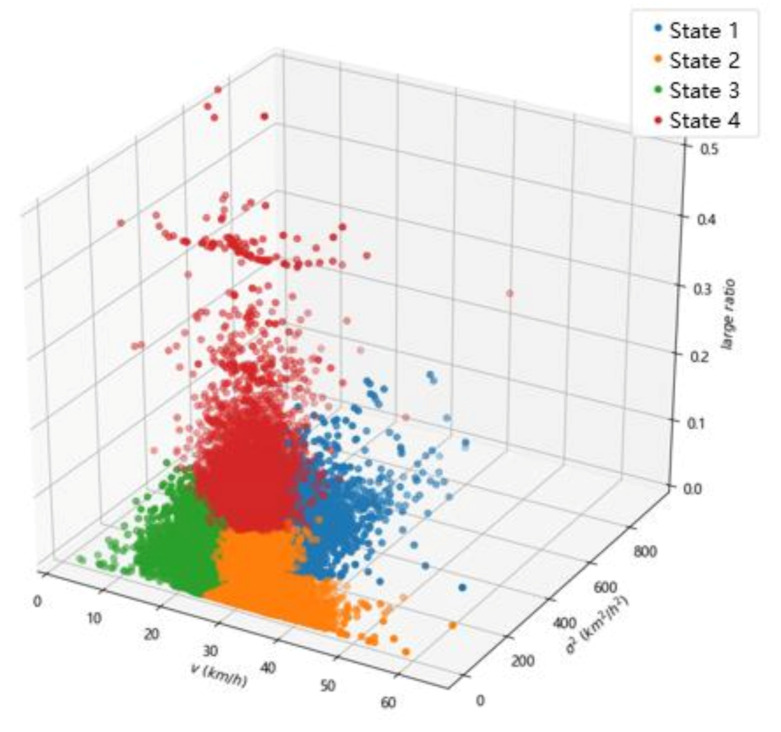
Clustering results of k=4

The following analyzes the results of traffic state clustering. Select the number of clusters k=4 to cluster the dataset, and the results obtained are shown in Figure 6.

**Figure 7:**
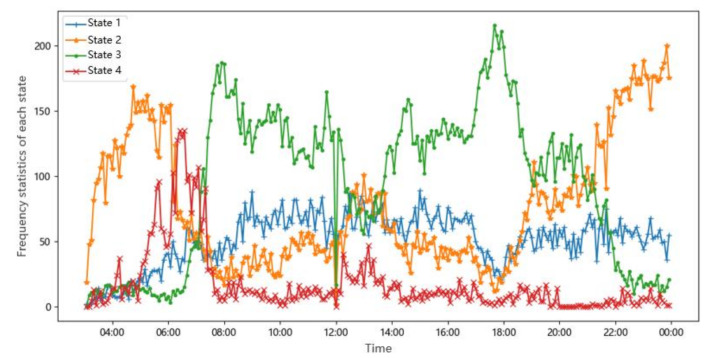
Frequency statistics of each state.

The mean value of the original indicator data contained in each state is analyzed after classification. The mean value of each indicator is shown in Table 3. We counted the times that each traffic state appears in all samples (i.e., the frequency of each state). Then, the frequency is regarded as the vertical axis, and time is regarded as the horizontal axis; the statistical results are shown in Figure 7. It can be seen from the figure that the four traffic states reached their peaks in different time periods. For example, the obvious characteristic of State 2 is that the traffic flow is low, and the roads are usually in this state at night. State 3 obviously occurs more frequently in the morning and evening rush hours. The more prominent characteristic of State 4 is that the large ratio is very high, and the frequency of State 4 occurs from 6 to 8 in the morning. This is basically consistent with the actual situation.

We compared the mean flow, mean speed, maximum speed and minimum speed of the four states to analyze the relative congestion degree of each state, and their mean speed variance was compared as an analysis of road safety, and finally, the comparison of the four mean large ratios was regarded as the degree of road stability. The greater the mean speed variance, the higher the road safety. The smaller the mean large ratio, the higher the road stability. Through the comparative analysis of the traffic state evaluation indicators under different states, the following conclusions can be drawn:

The mean traffic flow of State 1 (43.16 vec/5min) is significantly higher than that in State 2 and State 4, the maximum speed (48.51 km/h) and minimum speed (18.89 km/h) of State 1 are smaller than that in State 2 and State 4. Compared with State 3, State 1 has a smaller mean flow and a higher speed value. Therefore, State 1 ranks the third in terms of road patency; thus, we defined State 1 as a crowded state. At this time, the mean speed variance reached the highest (221.87); thus, the roads in State 1 are the least safe and rank the fourth in terms of safety. According to the mean large ratio of State 1 (3.56%), State 1 ranks the third in road stability. After analyzing and calculating the original data, we knew that the samples classified as State 1 accounted for 21.4% of the original samples. State 1 indicates that the road is in a crowded state, the road safety is poor, and there is a high safety risk. Therefore, State 1 is named crowded–unsafe–stable state.

State 2 has a low traffic flow (26.74 vec/5min), which is close to State 4, and reaches the highest mean speed (30.99 km/h), maximum speed (55.38 km/h), and minimum speed (25.12 km/h). Therefore, State 2 ranks the first in terms of road patency. In addition, State 2 has the smallest mean speed variance (73.95) and the smallest mean large ratio (1.78%), so State 2 ranks first in terms of road safety and road stability. After analyzing and calculating the original data, we knew that the samples classified as State 2 accounted for 32.13% of the original samples. This state is mainly concentrated in off-peak hours. It is concluded that the road is in an unblocked state in state 2, and the road safety and stability are high. State 2 is named unblocked–safe–stable state.

The mean traffic flow in State 3 (47.30 vec/5min) is the highest among the four traffic states, and the three speeds (mean speed 23.94 km/h, maximum speed 29.03 km/h, minimum speed 3.60 km/h) are also the lowest. Therefore, State 3 indicates that the road is blocked, and State 3 ranks the fourth in terms of the road patency. It can be seen from the mean speed variance (115.86) and the mean large ratio (3.51%) that State 3 ranks the third in road safety and the second in road stability. After analyzing and calculating the original data, it is found that the samples classified as State 3 accounted for 39.18% of the original samples. Thus, the state is mainly concentrated in peak hours. It is concluded that the road is blocked in State 3, and the road safety is not high. Thus, State 3 is named congested–unsafe-–stable state.

State 4 has the lowest mean traffic flow (24.25 vec/5min), which is close to the mean flow of State 2. However, the mean speed is still not the highest. We supposed that this situation is caused by the high large ratio. According to the mean speed (28.68 km/h), maximum speed (51.59 km/h), and minimum speed (11.82 km/h), it can be said that State 4 ranks the second in terms of road patency, but ranks the fourth in terms of road stability because it has the highest mean large ratio (16.62%). According to the mean speed variance (109.51), State 4 ranks the second in terms of road safety. After analyzing and calculating the original data, it is found that the samples classified as State 4 accounted for 7.29% of the original samples. This state occurs most frequently in the morning. It is concluded that State 4 indicates that the proportion of large vehicles on the road is high and the road stability is poor. In general, State 4 can be named unblocked–safe–unstable state.

**Figure 8:**
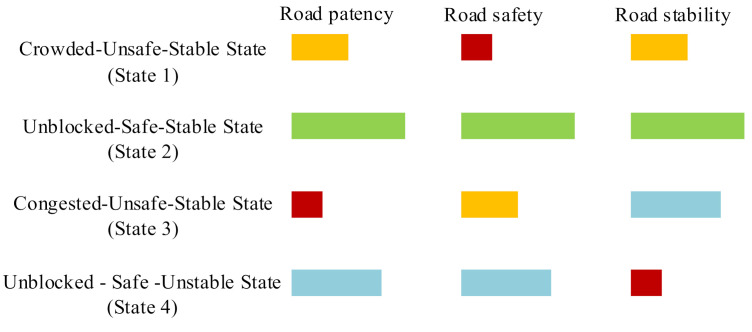
The ranking of the four states on each road evaluation indicator.

In this paper, we supposed that different people pay attention to different indicators (road patency, road safety, and road stability). For example, people who urgently need to reach their destination will pay more attention to road patency. Drivers driving trucks or coaches will pay more attention to road safety. Drivers driving small cars will hope that the road is stable and avoid meeting many large cars on the road. Therefore, we did not give the ranking of these four states. However, the ranking of the four states on each road evaluation indicator is described for people with different travel needs. As shown in Figure 8, the green box represents that the state ranks first in the corresponding evaluation indicator, the blue box represents the second, the yellow box represents the third, and the red box represents the fourth. It can be seen that the unblocked–safe–stable state (State 2) reached the first place in every indicator. The road in this state will be the best choice for travelers. However, when the road section that the traveler needs to pass does not have this state, travelers can choose road sections that show other traffic states based on more inclined road evaluation indicators.

### 3.3. Classifying Results and Visual Display

ROC space [40] defines the false positive rate (FPR) as the X axis and the true positive rate (TPR) as the Y axis. The TPR refers to the ratio of correctly judged as positive among all the actually positive samples. FPR represents the ratio of incorrectly judged as positive among all the actually negative samples. The perfect prediction is the (0,1) point in the ROC space. X = 0 means there are no false positives; Y = 1 means there are no false negatives, and the classification results that output by the classifier are all correct. In the multi-classification problem, one of the classes can be marked as a positive class, and the other classes are all marked as a negative class, the FPR and TPR of this class can be obtained, and the result can be plotted in the ROC space. In addition, the area under the ROC curve (AUC) is a better metric that can reflect the classification effect. The larger the value of the AUC, the better the classification effect, and the maximum value of AUC is 1.

**Figure 9:**
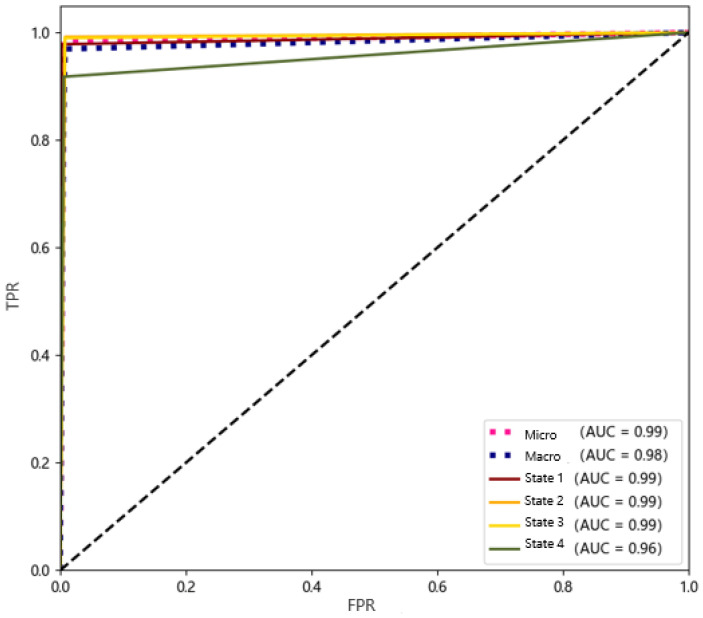
The receiver operating characteristic (ROC) curve of the classifier.

The training data of the classifier were derived from the K-means clustering results of the previous traffic flow data, and the clustering results were divided into a training set, a validation set, and a test set at a ratio of 80%–10%–10%. The result of the classifier on the test set is shown in Figure 9.

In the ROC space, the closer the point is to the upper left, the better the classification result. As can be seen from the figure, our classifier has achieved a good classification effect. In addition, the AUC of State 1, State 2, and State 3 are all 0.99, which is close to 1, indicating that the discrimination accuracy is relatively high. The AUC after macro-averaging is 0.98; the AUC after micro-averaging is 0.99. Comprehensive analysis shows that the FPR of the classification result is very low; the TPR is close to 1. We can draw that the probability of the classifier misjudgment is very low, and the existing samples can be accurately classified.

Finally, we discriminate and visually display the traffic state through the discrimination method proposed in this paper. We first access the database and obtain the road traffic state data from the front end. Based on the results of the traffic state discrimination, we visually display the distribution of traffic states in the road network through different colors and use circular graphs to visualize the proportions of varying traffic states.

**Figure 10:**
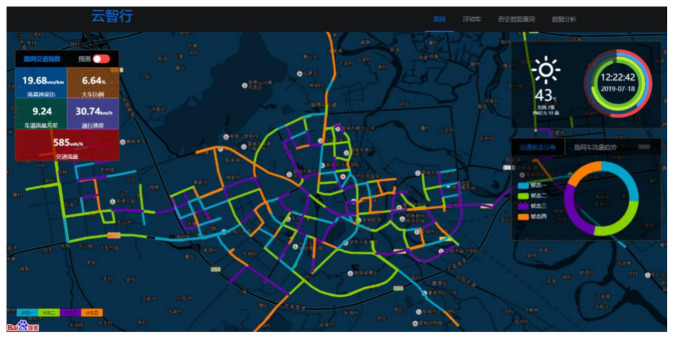
Comprehensive traffic state overview interface of the central city area.

Through the platform display example, it can be seen that in the urban road traffic state at this moment, State 3 accounts for the largest proportion, reaching 27.65%, and State 4 accounts for the smallest proportion, only 16.63%. We selected five traffic evaluation indicators: the traffic speed ratio, large ratio, road flow variance, traffic speed, and traffic flow to visually display the overall traffic state of the road network. The comprehensive traffic state overview interface of the central city area is shown in Figure 10.

## 4. Discussion

This paper first considers that the traffic state should not be represented only by a single indicator. Similar to the previous research, we chose the approach of multiple indicators for representation. However, although previous studies have adopted multiple indicators, they still only classify the traffic state according to the degree of congestion. We believe that the traffic state should not be represented only from whether the road is congested or not, so we chose the other two layers, including road safety and road stability, to judge the traffic state comprehensively. As in the previous study [10], we employed speed as an indicator of road congestion layer. Furthermore, time–mean–speed can be used as another embodiment of traffic density, which has been proved to be closely related to the state of traffic congestion in research [9]. In addition, the study [35] pointed out that road congestion becomes more serious, resulting in the deviation between the time–mean–speed and the space–mean–speed increases. According to the relationship between the two types of speed, we utilized the variance of the time–mean–speed observation value to express the deviation of the vehicle speed on the road, which can reflect the dispersion of the vehicle speed distribution. Thus, we applied this indicator as a parameter of the road safety layer. Existing studies have shown that the mixing of large vehicles will reduce the speed of conventional vehicles. Therefore, we chose the large ratio (LR) to evaluate the stability of the road.

After determining the three indicators, we need to represent the traffic state based on these data, that is to say, classify the traffic state according to these three indicators, and determine how many classes (traffic states) can represent different traffic conditions well. These classes should be able to comprehensively show the level of road congestion, road safety, and road stability. There should be no overlap between the different classes. Based on research [36], we found the superiority of the K-means algorithm in clustering. Therefore, this paper utilized the K-means algorithm to classify the traffic state. As we all know, the key of the K-Means algorithm lies in selecting the number of clusters (k), but determining the value of k based on artificial experience often fails to obtain the optimal clustering results. In order to solve this problem, some scholars have proposed that two indicators, including CH and SC, be used to discriminate the clustering results. Therefore, we supposed that if we set up multiple values of k and then compared the clustering effects through CH and SC, we can determine how many clusters can make our results optimal. Finally, after experiments, we divided the traffic state into four classes, each of which can comprehensively represent a traffic state.

There have been many studies for the discrimination of traffic state. Many methods based on SVM or ANN to discriminate and predict traffic state have been adopted. Since MLP has strong nonlinear mapping capabilities and can handle a large amount of complex data, we finally chose MLP to discriminate the traffic state, and the clustering result was used as the class label of our dataset. The experimental results also show that the employment of MLP to discriminate the traffic state has a very superior effect. However, there are still some problems during the experiment that can be further studied. In the clustering experiment, other algorithms or improved algorithms on the basis of K-Means can be further selected. In addition, the representation and discrimination of the traffic state are used as the basis of prediction, and we can further study how to predict the traffic state in real-time based on this paper in future work. This work can be used to predict the overall level of road congestion, road safety, and road stability in the future, and it will have profound significance for traffic control and travel planning.

## 5. Conclusions

Nowadays, traffic congestion is a problem that must be solved, and traffic safety is also the guarantee of people’s happy life. Thus, an accurate and comprehensive representation of traffic state is more and more important. However, existing studies usually only divide the road traffic state according to the road congestion state, which cannot reflect the state of road traffic at different layers. 

The main works of this paper are as follows. (1) In order to solve the problem of judging the traffic state only from the degree of congestion, we selected parameters from the layer of road congestion, road safety, and road stability. (2) Then we utilized the K-means clustering algorithm to classify and represent traffic states. The experimental results show that the traffic states can be divided into four classes to represent the current road congestion, safety, and stability. (3) In addition, previous researches have shown that ANN is effective in the field of traffic state discrimination. In this paper, a multi-layer perceptron (MLP) model was used to classify the traffic states, where the class labels are the four traffic states that we represented by the three indicators. The experimental results showed that MLP performs better as a traffic state classifier. The novel proposed model could be applied to traffic state analysis and prediction, traffic monitoring, and fleet management for various congestion scenarios in specific situations.

However, road traffic state always includes the discrimination of traffic state and the prediction of traffic state. Our work was not compared with other clustering algorithms, which is a major disadvantage. In the following research, we will try to use other clustering algorithms to cluster the traffic state. In addition, based on the conclusions and findings of this paper, the prediction of traffic state can be further explored in the future.

## Figures and Tables

**Table 1:** The main fields of the dataset.

FieldName	Description
DEVICEID	Device ID
FROMTIME	Statistical starting time
TOTIME	Statistical end time
INTERVAL	Statistics time interval
LANEID	Lane ID
COUNT	The count of vehicles in the interval
REGULARCOUNT	The count of regular vehicles in the interval
LARGECOUNT	The count of large vehicles in the interval
FLOW	Section conversion hourly flow
ARITHMETIC_AVERAGE_SPEED	Arithmetic average of speed
HARMONIC_AVERAGE_SPEED	Harmonic average of speed
TURN	Lane direction information

**Table 2:** Classification of the road section traffic state.

	Unblocked	Basically Unblocked	Lightly Congested	Moderately Congested	Severely Congested
Express Way	V>65	50<V≤65	35<V≤50	20<V≤35	V≤20
Trunk Road	V>40	30<V≤40	20<V≤30	15<V≤20	V≤15
Secondary Road and Branch Road	V>35	25<V≤35	15<V≤25	10<V≤15	V≤10

**Table 3:** Analysis of the mean value of the evaluation indicators for each state.

	State	1	2	3	4
Indicator					
Mean flow (vec/5min)		43.16	26.74	47.30	24.25
Mean speed (km/)		28.16	30.99	23.94	28.68
Maximum speed (km/h)		48.51	55.38	29.03	51.59
Minimum speed (km/h)		18.89	25.12	3.60	11.82
Mean speed variance		221.87	73.94	115.86	109.51
Mean large ratio		3.56%	1.78%	3.51%	16.62%

## References

[1] Barth M., Boriboonsomsin K. (2008). Real-World Carbon Dioxide Impacts of Traffic Congestion. Transp. Res. Rec..

[2] Yuan Y. Application of Intelligent Technology in Urban Traffic Congestion. Proceedings of the 2020 International Conference on Computer Engineering and Application (ICCEA).

[3] Zahid M., Chen Y., Jamal A., Memon M.Q. (2020). Short Term Traffic State Prediction via Hyperparameter Optimization Based Classifiers. Sensors.

[4] Park H.-C., Kim D.-K., Kho S.-Y. (2018). Bayesian Network for Freeway Traffic State Prediction. Transp. Res. Rec..

[5] Nanthawichit C., Nakatsuji T., Suzuki H. (2003). Application of Probe-Vehicle Data for Real-Time Traffic-State Estimation and Short-Term Travel-Time Prediction on a Freeway. Transp. Res. Rec. J..

[6] Wang B., Sun J., Wang W., Xu Z., Tian T., Wang Y., Wei J. Real Time Detection of Traffic Signal Running State and Remote Alarm for Fault Information at Road Intersection. Proceedings of the 2018 24th International Conference on Automation and Computing (ICAC), Newcastle upon Tyne.

[7] Chen Z., Cai H., Zhang Y., Wu C., Mu M., Li Z., Sotelo M.A. (2019). A novel sparse representation model for pedestrian abnormal trajectory understanding. Expert Syst. Appl..

[8] Chen Z.J., Wu C.Z., Zhang Y.S., Huang Z., Jiang J.F., Lyu N.C., Ran B. (2016). Vehicle Behavior Learning via Sparse Reconstruction with l2-lp Minimization and Trajectory Similarity. IEEE Trans. Intell. Transp. Syst..

[9] Khan S.M., Dey K.C., Chowdhury M. (2017). Real-Time Traffic State Estimation with Connected Vehicles. IEEE Trans. Intell. Transp. Syst..

[10] Shi W., Liu Y. (2010). Real-time urban traffic monitoring with global positioning system-equipped vehicles. IET Intell. Transp. Syst..

[11] Tao S., Manolopoulos V., Rodriguez S., Rusu A. (2012). Real-Time Urban Traffic State Estimation with A-GPS Mobile Phones as Probes. J. Transp. Technol..

[12] Hu Q., Deng W., Sun X. (2015). The comprehensive measure model for urban traffic congestion based on value function. J. Southeast Univ..

[13] Seo T., Kusakabe T., Asakura Y. (2015). Estimation of Flow and Density Using Probe Vehicles with Spacing Measurement Equipment. Transp. Res. Part C Emerg. Technol..

[14] Wan Q., Peng G., Li Z., Inomata F.H.T. (2020). Spatiotemporal trajectory characteristic analysis for traffic state transition prediction near expressway merge bottleneck. Transp. Res. Part C Emerg. Technol..

[15] Antoniou C., Koutsopoulos H.N., Yannis G. (2013). Dynamic data-driven local traffic state estimation and prediction. Transp. Res. Part C Emerg. Technol..

[16] Wang Y., Papageorgiou M. (2005). Real-time freeway traffic state estimation based on extended Kalman filter: A general approach. Transp. Res. Part B Methodol..

[17] Wang P.-W., Yu H.-B., Xiao L., Wang L. (2017). Online Traffic Condition Evaluation Method for Connected Vehicles Based on Multisource Data Fusion. J. Sens..

[18] Xu D., Wang Y., Peng P., Beilun S., Deng Z., Guo H. (2018). Real-time road traffic state prediction based on kernel-KNN. Transp. A Transp. Sci..

[19] Zhan X., Li R., Ukkusuri S.V. (2020). Link-based traffic state estimation and prediction for arterial networks using license-plate recognition data. Transp. Res. Part C Emerg. Technol..

[20] Cheng Z., Wang W., Lu J., Xing X. (2020). Classifying the traffic state of urban expressways: A machine-learning approach. Transp. Res. Part A Policy Pract..

[21] Quek C., Pasquier M., Lim B. (2006). POP-TRAFFIC: A Novel Fuzzy Neural Approach to Road Traffic Analysis and Prediction. IEEE Trans. Intell. Transp. Syst..

[22] Stutz C., Runkler T.A. (2002). Classification and prediction of road traffic using application-specific fuzzy clustering. IEEE Trans. Fuzzy Syst..

[23] Thomas K., Dia H. A Neural Network Model for Arterial Incident Detection Using Probe Vehicle and Loop Detector Data. https://www.researchgate.net/publication/43483712_A_neural_network_model_for_arterial_incident_detection_using_probe_vehicle_and_loop_detector_data.

[24] Xu D., Wei C., Peng P., Xuan Q., Guo H. (2020). GE-GAN: A novel deep learning framework for road traffic state estimation. Transp. Res. Part C Emerg. Technol..

[25] Qin P., Xu Z., Yang W., Liu G., Li J. (2018). Real-Time Road Traffic State Prediction Based on SVM and Kalman Filter. Wireless Sensor Networks.

[26] Min Z., Yanlei L., Dihua S., Senlin C. Highway Traffic Abnormal State Detection Based on PCA-GA-SVM Algorithm. Proceedings of the 2017 29th Chinese Control and Decision Conference.

[27] Xue J., Van Gelder P., Reniers G., Papadimitriou E., Wu C. (2019). Multi-attribute decision-making method for prioritizing maritime traffic safety influencing factors of autonomous ships’ maneuvering decisions using grey and fuzzy theories. Saf. Sci..

[28] Xue J., Wu C., Chen Z., Van Gelder P., Liang X. (2019). Modeling human-like decision-making for inbound smart ships based on fuzzy decision trees. Expert Syst. Appl..

[29] Hawas Y.E. (2007). A fuzzy-based system for incident detection in urban street networks. Transp. Res. Part C Emerg. Technol..

[30] Yuan F., Cheu R.L. (2003). Incident detection using support vector machines. Transp. Res. Part C Emerg. Technol..

[31] Ritchie S.G., Cheu R.L. (1993). Simulation of Freeway Incident Detection Using Artificial Neural Networks. Transp. Res. Part C Emerg. Technol..

[32] Chen Z., Jiang Y., Sun D., Liu X. (2019). Discrimination and Prediction of Traffic Congestion States of Urban Road Network Based on Spatio-Temporal Correlation. IEEE Access.

[33] Dong C., Shao C., Richards S.H., Han L.D. (2014). Flow rate and time mean speed predictions for the urban freeway network using state space models. Transp. Res. Part C Emerg. Technol..

[34] Raj J., Bahuleyan H., Vanajakshi L.D., Patil G.R., Mathew T.V., Rao K.V.K. (2016). Application of data mining techniques for traffic density estimation and prediction. Proceedings of the International Conference on Transportation Planning and Implementation Methodologies for Developing Countries.

[35] Han E., Kim S.B., Rho J.H., Yun I., Ajou University (2016). Comparison of the Methodologies for Calculating Expressway Space Mean Speed Using Vehicular Trajectory Information from a Radar Detector. J. Korea Inst. Intell. Transp. Syst..

[36] Yu Y., Trouvé A. A non-linear K-means algorithm and its application to unsupervised clustering. Proceedings of the 6th International Conference on Signal Processing 2002 ICOSP-02.

[37] Mitra S., Pal S. (1995). Fuzzy multi-layer perceptron, inferencing and rule generation. IEEE Trans. Neural Netw..

[38] Caliński T., Harabasz J. (1974). A dendrite method for cluster analysis. Commun. Stat..

[39] Stephens C.R., Sukumar R. (2006). An Introduction to Data Mining.

[40] Fawcett T. (2006). An introduction to ROC analysis. Pattern Recognit. Lett..

